# Preparation, characterization and luminescence properties of core–shell ternary terbium composites SiO_2(600)_@Tb(MABA-Si)•L

**DOI:** 10.1098/rsos.171655

**Published:** 2018-03-14

**Authors:** Yang-Yang Ma, Wen-Xian Li, Yu-Shan Zheng, Jin-Rong Bao, Yi-Lian Li, Li-Na Feng, Kui-Suo Yang, Yan Qiao, An-Ping Wu

**Affiliations:** 1 College of Chemistry and Chemical Engineering, Inner Mongolia University, Hohhot 010021, People's Republic of China; 2 Inner Mongolia Autonomous Region food inspection test center, Hohhot 010021, People's Republic of China

**Keywords:** SiO_2(600)_@Tb(MABA-Si)·L core–shell materials, silylated *m*-aminobenzoic acid (MABA-Si), dipy and phen, ternary terbium complex, luminescence, lifetime

## Abstract

Two novel core–shell structure ternary terbium composites SiO_2(600)_@Tb(MABA-Si)·L(L:dipy/phen) nanometre luminescence materials were prepared by ternary terbium complexes Tb(MABA-Si)·L_2_·(ClO_4_)_3_·2H_2_O shell grafted onto the surface of SiO_2_ microspheres. And corresponding ternary terbium complexes were synthesized using (CONH(CH_2_)_3_Si(OCH_2_CH_3_)_3_)_2_ (denoted as MABA-Si) as first ligand and L as second ligand coordinated with terbium perchlorate. The as-synthesized products were characterized by means of IR spectra, ^1^HNMR, element analysis, molar conductivity, SEM and TEM. It was found that the first ligand MABA-Si of terbium ternary complex hydrolysed to generate the Si–OH and the Si–OH condensate with the Si–OH on the surface of SiO_2_ microspheres; then ligand MABA-Si grafted onto the surface of SiO_2_ microspheres. The diameter of SiO_2_ core of SiO_2(600)_@Tb(MABA-Si)·L was approximately 600 nm. Interestingly, the luminescence properties demonstrate that the two core–shell structure ternary terbium composites SiO_2(600)_Tb(MABA-Si)·L(dipy/phen) exhibit strong emission intensities, which are 2.49 and 3.35 times higher than that of the corresponding complexes Tb(MABA-Si)·L_2_·(ClO_4_)_3_·2H_2_O, respectively. Luminescence decay curves show that core–shell structure ternary terbium composites have longer lifetime. Excellent luminescence properties enable the core–shell materials to have potential applications in medicine, industry, luminescent fibres and various biomaterials fields.

## 1. Introduction

There has been extensive arousing of interest in core–shell structure nanomaterials because of the interesting properties that can be employed in optical, electrical, magnetic and biological applications [1–7]. It can be found that a nanometre material was covered with another nanometre material by chemical bond or another affinity. The functionality of their properties is a critical factor that promotes the development of nanocomposites materials [8–11]. In particular, luminescence applications of rare earth core–shell nanomaterials are very attractive because of their superior luminescence intensity. Nowadays, they are used or are being tested for use in such fields as medicine, industry, luminescent fibres and various biomaterials [12–17]. Now, such rare earth core–shell nanomaterials have attracted considerable attention due to possessing high photostability and thermal stability that they have potential applications in luminescent areas [18–20]. Rare earth core–shell nanometre composite was a research subject that would be a good choice for improving the luminescence intensity and lifetime.

In the rare earth core–shell nanomaterials field, SiO_2_ as the core was popular in recent years. SiO_2_ microspheres are regarded as ideal core materials with several advantages [21]. First, SiO_2_ possesses strong physical stability, which can fix the organic functional groups. Furthermore, SiO_2_ can be obtained at room temperature because of convenient reaction conditions. SiO_2_ is considered an ideal low-cost material that has previously been used for various core microsphere. SiO_2_ core–shell nanomaterials can be obtained by covalent bonds. Good results have been obtained with functionalized siloxanes, because they form strong covalent bonds with most SiO_2_ surfaces due to the presence of hydroxyl groups. The large number of commercially available trialkoxysilanes with various functional groups offers unique possibilities for the task-specific surface modification of SiO_2_ microsphere. For example, MABA-Si can act as a ‘bridge molecule’ that connects with rare earth and SiO_2_ to enhance physical stability and decrease the energy loss. In this way, core–shell structure materials connected by covalent bond are stable so that the covalent bond is difficult to break. As a result, these kinds of core-shell structure nanometre composites have high luminescence intensity. Therefore, these materials have become an active field of research due to their physical and chemical properties, as well as their potential application. So far, silica has been investigated in the field of DNA, fluorescent probes and sensing [22,23].

In our work, the synthesis and structure as well as luminescence properties of the SiO_2(600)_@Tb(MABA-Si)·L composites that have SiO_2_ as the core and ternary terbium complex as the shell was studied. Functionalized organosilane (denoted as MABA-Si) not only graft onto the SiO_2_ surface by covalently bonding but also coordinates with rare earth ions (Tb^3+^). To improve the luminescent performance of rare earth ions (Tb^3+^), another kind of small-molecule ligand with a conjugate system (phenanthroline (phen) or dipyridine (dipy)) was introduced, not only meeting the rare earth ions (Tb^3+^) coordination number but also having more efficient energy absorption and energy transfer. Terbium core–shell composites possess high luminescence intensity and long lifetime. The study of core–shell structure terbium luminescent nanomaterial is more meaningful to dispose of the potential application.

## 2. Material and methods

### 2.1. Chemicals

The starting materials for the preparation of SiO_2_ were tetraethoxysilane (TEOS), ammonium hydroxide, water and anhydrous ethanol. Tb_4_O_7_ (99.999%) was dissolved in perchloric acid to prepare Tb(ClO_4_)_3_·nH_2_O. 3-(triethoxysilyl)-propyl isocyanate (TEPIC, 96%, Aldrich), pure phen, dipy, *m*-aminobenzoic acid were also used. All other chemical reagents were analytical grade.

### 2.2. Physical measurements

Elemental analysis was taken with a HANAU analyser. Infrared spectra (IR, *υ* = 4000–400 cm^−1^) was obtained by a Nicolet NEXUS-670 FT-IR spectrophotometer, which was determined by the KBr pellet technique. Luminescence excitation and emission spectra were performed on FLS980 spectrophotometer at room temperature (slit width was 0.5 nm). Luminescence lifetime measurements were recorded by FLS980 Combined Steady State and Lifetime Spectrometer (slit width was 0.5 nm). Scanning electron microscope (SEM) images were recorded with a Hitachi S-4800. Transmission electron microscopy (TEM) and energy dispersive X-ray spectroscopy were performed on a FEI Tecnai F20 operated at 200 kV. Conductivity measurement was made by using 1 × 10^3^ mol l^−1^ solution in dimethylformamide (DMF) on a DDS-11D conductivity metre at room temperature. The terbium content of the complex was measured by EDTA titration using xylenol-orange as an indicator.

### 2.3. Synthesis of the ternary terbium complexes

#### 2.3.1. Synthesis of silylated *m*-aminobenzoic acid (MABA-Si)

**Figure 1.**
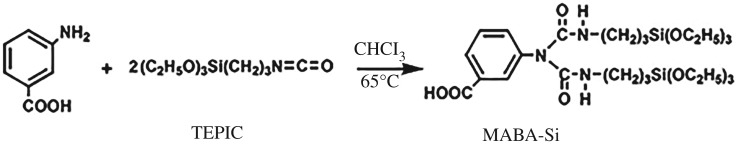
The synthesis scheme of MABA-Si.

The synthesis scheme of MABA-Si is shown in figure 1. *m*-Aminobenzoic acid (2 mmol) dissolved in 40 ml chloroform, 4 mmol 3-(triethoxysilyl)-propyl isocyanate was added dropwise into above solution with stirring at 60°C for 12 h. The obtained white precipitate was isolated by centrifugation, washed with water and ethanol and dried in an oven overnight at 60°C [24,25]. Yield: 40%. The resultant sample was characterized by ^1^HNMR and elemental analysis. ^1^HNMR: *δ*0.56 ppm(4H), *δ*1.04–1.51 ppm(18H), *δ*2.93 ppm(4H), *δ*3.33–3.47 ppm(12H), *δ*3.73 ppm(4H), *δ*7.43–7.57 ppm(2H) and *δ*12.4 ppm(1H). Anal. Calcd. of C_28_H_49_N_3_O_10_Si_2_ (M = 631 g mol^−1^): C, 51.35%; H, 7.76%; N, 6.66%; found: C, 50.80%; H, 7.82%; N, 6.55%. m.p.: 46–48°C.

#### 2.3.2. Synthesis of ternary terbium complexes of Tb(MABA-Si)·L_2_·(ClO_4_)_3_·2H_2_O

MABA-Si (1 mmol) and 2 mmol phen (or dipy) were dissolved in 10 ml anhydrous ethanol, and then 1 mmol Tb(ClO_4_)_3_·nH_2_O was added in the above solution with stirring at 60°C for 2 h, the Tb^3+^: MABA-Si: L molar ratio of 1 : 1 : 2. The white precipitate was isolated by centrifugation and washed with anhydrous ethanol. The elemental analysis and molar conductivities of the products were measured (table 1). Anal. Calcd. of Tb(MABA-Si)·dipy_2_·(ClO_4_)_3_·2H_2_O (M = 1509.26 g mol^−1^): C, 38.33; H, 6.44; N, 4.79; Tb, 10.21; found: C, 37.40; H, 6.49; N, 4.57; Tb, 10.53; and Anal. Calcd. of Tb(MABA-Si)·phen_2_·(ClO_4_)_3_·H_2_O (M = 1557.7 g mol^−1^): C, 40.02; H, 6.37; N, 4.28; Tb, 9.85, found: C, 39.90; H, 6.29; N, 4.43; Tb, 10.02. The corresponding molar conductivities were 148 S cm^2^ mol^−1^ and 160 S cm^2^ mol^−1^. The ternary terbium complexes formulated 1 : 2 electrolytes [26].

**Table 1.** Composition (%) and molar conductivities (S cm^2^ mol^−1^) of ternary terbium complexes. Calculated value in brackets.

ternary terbium complexes	M(g mol^−1^)	C	N	H	RE	*λ*m
Tb(MABA-Si)·dipy_2_·(ClO_4_)_3_·2H_2_O	1509.26	38.33 (37.40)	6.44 (6.49)	4.79 (4.57)	10.21 (10.53)	148
Tb(MABA-Si)·phen_2_·(ClO_4_)_3_·2H_2_O	1557.78	40.02 (39.90)	6.37 (6.29)	4.28 (4.43)	9.85 (10.02)	160

### 2.4 Preparations of core–shell structure ternary terbium composites SiO_2(600)_@Tb(MABA-Si)·L

#### 2.4.1. Synthesis of SiO_2_ monodisperse

Spherical SiO_2(600)_ was prepared by outstanding Stober method [27]. A total of 50 ml anhydrous ethanol, 2.6 ml ammonium hydroxide, 5 ml tetraethyl orthosilicate and 15 ml water were mixed with stirring for 6 h at room temperature (table 2). A white silica colloidal suspension was formed. The solid product was washed thoroughly with water and anhydrous ethanol [28].

**Table 2.** The volume of materials to prepare silica and reaction time.

EtOH (ml)	H_2_O (ml)	TEOS	NH_3_H_2_O (ml)	time (h)	size (nm)
50	15	5.0	2.6	5	600

#### 2.4.2. Synthesis of SiO_2(600)_@MABA-Si

To make MABA-Si graft onto SiO_2_ microspheres by the Si–O–Si bond, the SiO_2_ microspheres were activated with ammonium hydroxide. SiO_2_ (0.1 g) was dissoved in 10 ml water and 10 ml ethanol mixture solution. A certain amount of ammonium hydroxide was added into the above solution to adjust the pH to 9.2 with stirring for 12 h. The precipitate was washed with water and anhydrous ethanol three times. The obtained activated SiO_2_ microspheres and 0.2 g ligand MABA-Si were redissolved in 20 ml ethanol solution. The mixture solution was dispersed for 10 min by ultrasonication, then 10 ml water was added drop by drop. The above mixture solution was stirred for 10 h. The obtained precipitate was separated by centrifugation, washed with water and ethanol and dried in an oven at 60°C. As a result, MABA-Si was grafted onto SiO_2_ core by formation of Si–O–Si bond, SiO_2(600)_@MABA-Si was obtained.

#### 2.4.3. Synthesis SiO_2(600)_@Tb(MABA-Si)·L

Core–shell structure ternary terbium composite SiO_2(600)_@Tb(MABA-Si)·dipy was prepared as follows: 0.1 g SiO_2(600)_@MABA-Si and 0.2 g dipy were dissolved in 10 ml anhydrous ethanol, then the 5 ml anhydrous ethanol solution of 0.1 g Tb(ClO_4_)_3_·nH_2_O was added into the above mixture solution. The mixture solution was stirred at room temperature for 12 h, then the white precipitate was obtained. The obtained precipitate was separated by centrifugation, washed with distilled water and ethanol and dried in an oven at 60°C. The core–shell structure ternary terbium composite of SiO_2(600)_@Tb(MABA-Si)·dipy was synthesized successfully. The synthesis procedure for SiO_2(600)_@Tb(MABA-Si)·phen was similar to that of SiO_2(600)_@Tb(MABA-Si)·dipy except dipy was replaced by phen.

## 3. Results and discussion

### 3.1. IR spectra

#### 3.1.1. IR spectra of ternary terbium complexes

**Figure 2.**
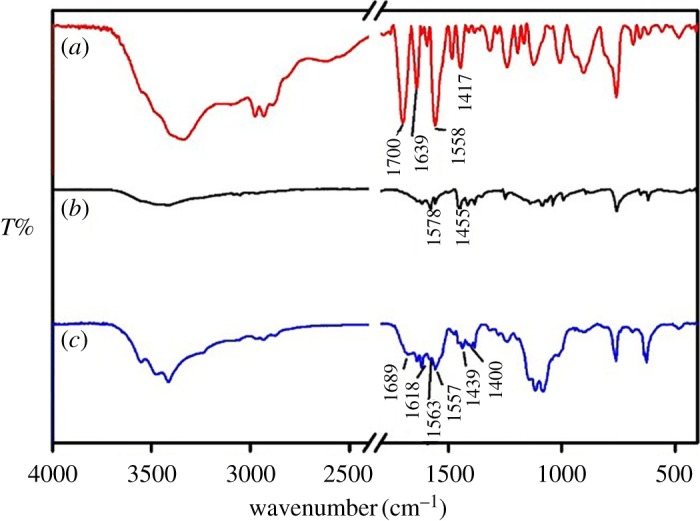
IR spectra of the MABA-Si (*a*), dipy (*b*) and Tb(MABA-Si)·dipy_2_·(ClO_4_)_3_·2H_2_O (*c*).

Figure 2*a–c* shows the IR spectra of MABA-Si, dipy and Tb(MABA-Si)·dipy_2_·(ClO_4_)_3_·2H_2_O. In the spectrum of MABA-Si, the characteristic peaks located at 1639 cm^−1^ (*ν*_C=O_) and 1558 cm^−1^ (*δ*_NH_) were attributed to stretching vibration and bending vibration of –CONH– (figure 2*a*). The characteristic absorption of amide group (–CONH–) suggested that MABA-Si has been successfully synthesized by the amidation reaction with MABA and 3-(triethoxysilyl)-propyl isocyanate. The characteristic peaks located at 1700 and 1417 cm^−1^ belonged to stretching vibration and bending vibration of –COOH. Figure 2*b* shows IR spectrum of dipy. The stretching vibration of C = N appeared at 1578 and 1455 cm^−1^. Futhermore, in the IR spectrum of ternary terbium complex Tb(MABA-Si)·dipy_2_·(ClO_4_)_3_·2H_2_O (figure 2*c*), the stretching vibration and bending vibration of the –COOH were red-shifted to 1689 and 1400 cm^−1^. In addition, the stretching vibration and bending vibration of –CONH– groups were shifted to 1618 cm^−1^ and 1557 cm^−1^, respectively. It indicated that MABA-Si coordinated with the Tb^3+^ ions by carboxylic group and amide groups [29–33]. The stretching vibration of C = N shifted low wavenumber, which appeared at 1557 and 1439 cm^−1^ in the ternary terbium complex Tb(MABA-Si)·dipy_2_·(ClO_4_)_3_·2H_2_O. The stretching vibration of C = N had obvious red shift, which showed that Tb^3+^ ions coordinated with two nitrogen atoms of dipy [34].

**Figure 3:**
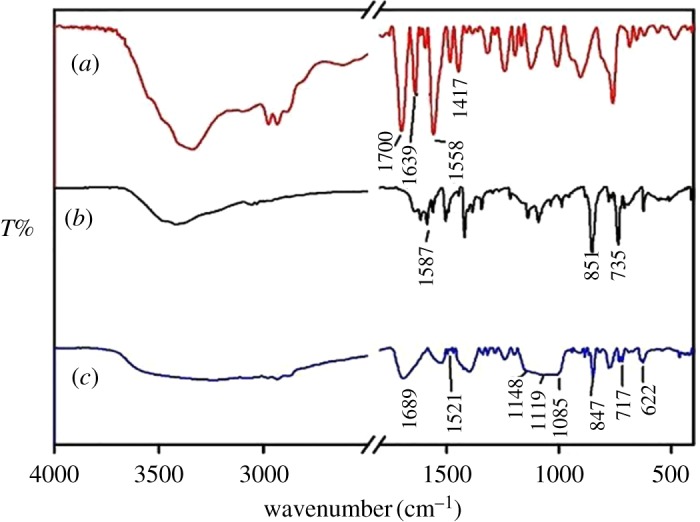
IR spectra of the MABA-Si (*a*), phen (*b*) and Tb(MABA-Si)·phen_2_·(ClO_4_)_3_·2H_2_O (*c*).

The IR spectra of MABA-Si, phen, Tb(MABA-Si)·phen_2_·(ClO_4_)_3_·2H_2_O are shown in figure 3*a–c*. Comparing the spectrum of Tb(MABA-Si)·phen_2_·(ClO_4_)_3_·2H_2_O with MABA-Si, the characteristic absorption peaks of carboxylic group appeared at 1689 cm^−1^, indicating that the carbonyl group of MABA-Si was coordinated with Tb^3+^ ions. The stretching vibration C = N group located at 1587 cm^−1^, bending vibration of C–H located at 735 and 851 cm^−1^ in the IR spectrum of phen (figure 3*b*). In the IR spectrum of ternary terbium complex Tb(MABA-Si)·phen_2_·(ClO_4_)_3_·2H_2_O (figure 3*c*), *ν*_C=N_ red-shifted to 1521 cm^−1^, *δ*_C–H_ red-shifted to 717 cm^−1^ and 847 cm^−1^, respectively. It displayed that the Tb^3+^ ions coordinated with double nitrogen atoms of phen [35].

In addition, the characteristic absorption peaks of perchlorate group can be found in the ternary terbium complex (figure 3*c*).The characteristic absorption peaks of perchlorate groups appeared around at 1148 cm^−1^, 1119 cm^−1^, 1085 cm^−1^, 622 cm^−1^, which indicated that perchlorate was involved in the coordination. One perchlorate group bonded with the Tb^3+^ ion, which corresponded with the results of the molar conductivity. Based on the literature, the vibration of perchlorate group just appeared at 1090 and 623 cm^−1^, which demonstrated that perchlorate group is responsible for Td symmetry, and perchlorate group did not coordinate with Tb^3+^ ions. When perchlorate coordinated with Tb^3+^ ions, the perchlorate appeared at approximately 1145 cm^−1^, 1115 cm^−1^, 1079 cm^−1^, 925 cm^−1^ and 627 cm^−1^; it was assigned to the C_2v_ symmetry [36,37]. It showed that three perchlorates of ternary terbium complexes were not completely Td symmetry. Some of them were C_2v_ symmetry which indicated that perchlorate was involved in the coordination.

#### 3.1.2. IR spectra of core–shell structure ternary terbium composites SiO_2(600)_@Tb(MABA-Si)·L

**Figure 4.**
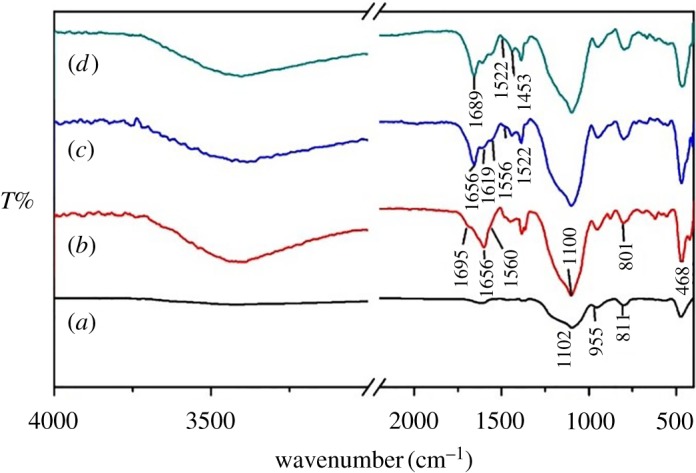
IR spectra of SiO_2_ (*a*), SiO_2(600)_@MABA-Si (*b*), SiO_2(600)_@Tb(MABA-Si)·phen (*c*) and SiO_2(600)_@Tb(MABA-Si)·dipy (*d*).

Figure 4*a–d* gives IR spectra of SiO_2_, SiO_2(600)_@MABA-Si, SiO_2(600)_@Tb(MABA-Si)·phen and SiO_2(600)_@Tb(MABSi)·dipy. In the spectrum of SiO_2_, the characteristic absorption peaks of Si–O–Si located at 1102 cm^−1^, and Si–OH was identified at 955 cm^−1^ (figure 4*a*). Thus, the Si–OH groups were predominantly adsorbed on the surfaces of SiO_2_. Figure 4*b* shows the IR spectrum of SiO_2(600)_@MABA-Si. The absorption peak located at 1695 cm^−1^ was attributed to stretching vibration of –COOH. The characteristic peaks located at 1656 cm^−1^ and 1560 cm^−1^ belonged to stretching vibration of –CONH– group of SiO_2(600)_@(MABA-Si). The broad bands located at approximately 1100 cm^−1^ and 801 cm^−1^ were assigned to the asymmetric stretching vibration and symmetric stretching vibration of Si–O–Si band, respectively. Furthermore, the peak of Si–O–Si intensity is greatly enhanced, which should result from the MABA-Si grafting onto the SiO_2_. It displays that the first ligand MABA-Si generates the Si–OH, and the Si–OH condensate with the Si–OH on the surface of SiO_2_ microspheres. In the IR spectrum of SiO_2(600)_@Tb(MABA-Si)·phen (figure 4*c*), the characteristic absorption peak of carboxylic group ─COOH appeared at 1656 cm^−1^. Furthermore, the –CONH– characteristic bands appeared at 1556 cm^−1^, indicating that carbonyl group and amide group were coordinated with Tb^3+^ ions. The peak that appeared at 1522 cm^−1^ was ascribed to stretching vibration of nitrogen atoms of phen in SiO_2(600)_@Tb(MABA-Si)·phen. It showed an obvious red shift compared with free phen (figure 3*a*), which proved that phen successfully coordinated with the Tb^3+^ ions. The IR spectrum of MABA-Si in SiO_2(600)_@Tb(MABA-Si)·dipy (figure 4*d*) is similar to that in SiO_2(600)_@Tb(MABA-Si)·phen. The characteristic peaks of dipy that appeared at 1522 cm^−1^ and 1453 cm^−1^ showed that dipy successfully coordinated with the Tb^3+^ ions. IR spectra showed that the ternary terbium complexes formed on the surface of SiO_2_ microspheres. The core–shell ternary terbium composites SiO_2(600)_@Tb(MABA-Si)·L were synthesized.

### 3.2. The TEM and SEM of core–shell structure ternary terbium composites SiO_2(600)_@Tb(MABA-Si)·L

**Figure 5.**
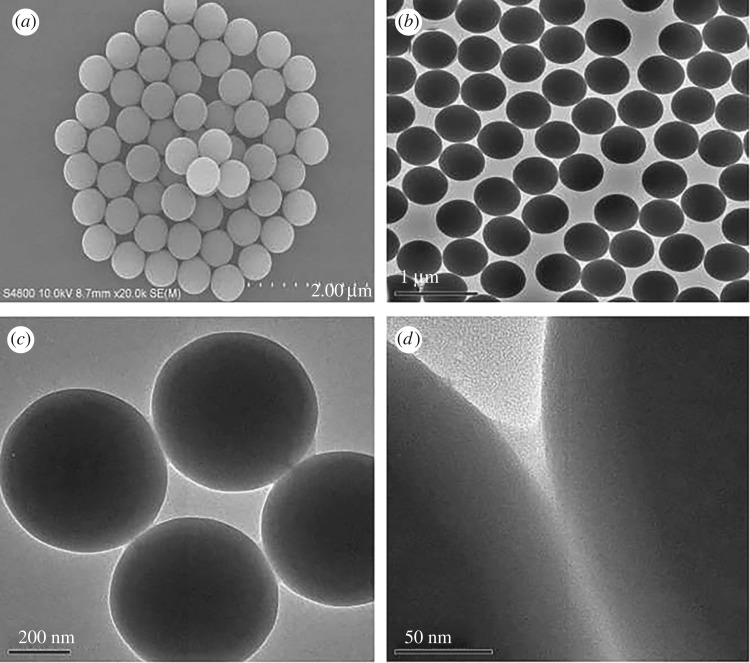
The SEM of SiO_2_ (*a*), TEM of SiO_2_ (*b*) and SiO_2(600)_@MABA-Si (*c*,*d*).

**Figure 6.**
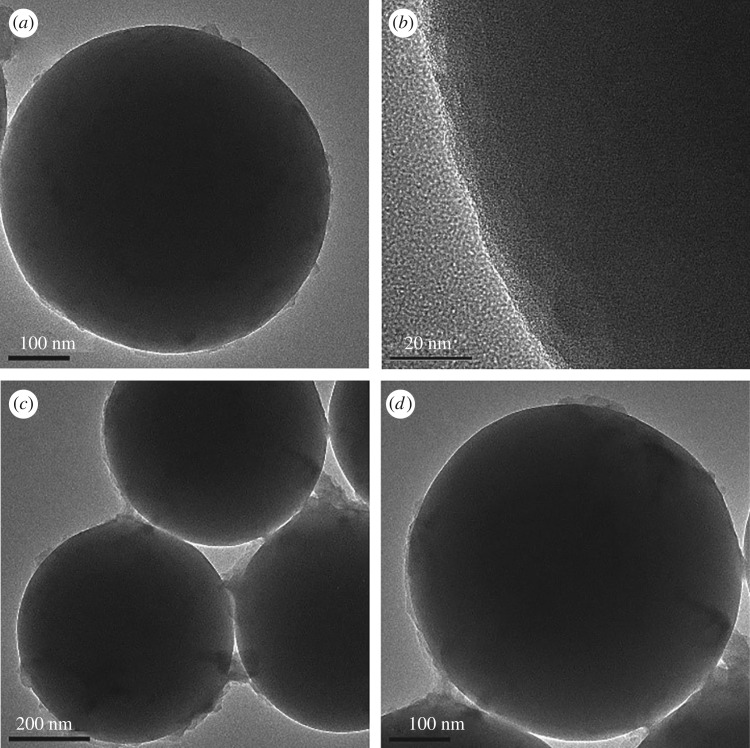
The TEM images of core–shell structures SiO_2(600)_@Tb(MABA-Si)·phen (*a*,*b*), SiO_2(600)_@Tb(MABA-Si)·dipy (*c*,*d*).

**Figure 7.**
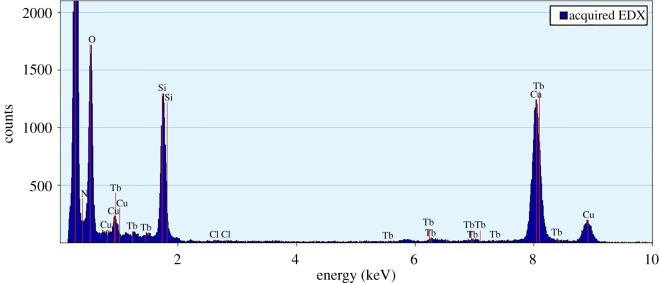
The EDX spectrum of core–shell structure ternary terbium composites.

**Figure 8.**
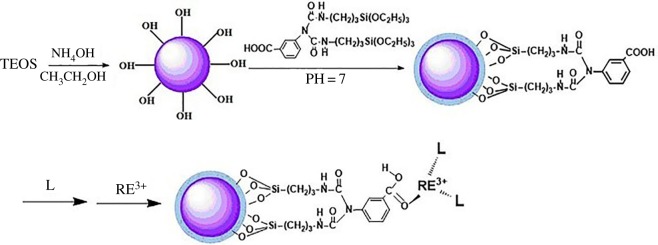
The formation mechanism of core–shell structures SiO_2(600)_@Tb(MABA-Si)·L.

Scanning electron microscopy (SEM) and transmission electron microscopy (TEM) were used to monitor the fabrication of the as-synthesized products. Figure 5 shows the SEM (*a*), TEM of the SiO_2_ (*b*) and TEM of the SiO_2(600)_@MABA-Si (*c*, *d*). From figure 5*a* and *b*, we can observe that the as-formed SiO_2_ core has a smooth surface, and the diameter is approximately 600 nm. The typical TEM images of SiO_2(600)_@MABA-Si are shown in figure 5*c,d*, which reveals that SiO_2(600)_@(MABA-Si) has a core–shell structure and presents a uniform spherical morphology with thin layer. The images (figure 6*a–d*) of SiO_2(600)_@Tb(MABA-Si)·phen and SiO_2(600)_@Tb(MABA-Si)·dipy show that the surface of SiO_2(600)_@Tb(MABA-Si)·L becomes much rougher and has an obvious layer, which might be caused by the ternary terbium complexes grafted onto the surface of SiO_2_ core. Figure 6*b* indicates that the Tb(MABA-Si)·dipy and Tb(MABA-Si)·phen surface layer has a diameter of approximately 5 nm. EDX analysis of SiO_2(600)_@Tb(MABA-Si)·L (figure 7) confirms the existence of Cl, Tb, N, O and Si, which gives experimental evidence for the existence of the core–shell structure ternary terbium composites. The formation mechanism of core–shell structure composite is inferred (figure 8). In this process, Si–O–Si chemical bond can be formed by hydrolysis-polycondensation method from ethoxy of MABA-Si and hydroxyl groups of the SiO_2_ surface. Then the carboxyl oxygen atoms of MABA-Si groups can be coordinated with Tb^3+^ ions. It is quite possible that the ternary terbium complex grafted onto the surface of the SiO_2_ at the early stage of the reactions, due to the synthesized Si–O–Si band.

### 3.3. Luminescence properties

**Figure 9.**
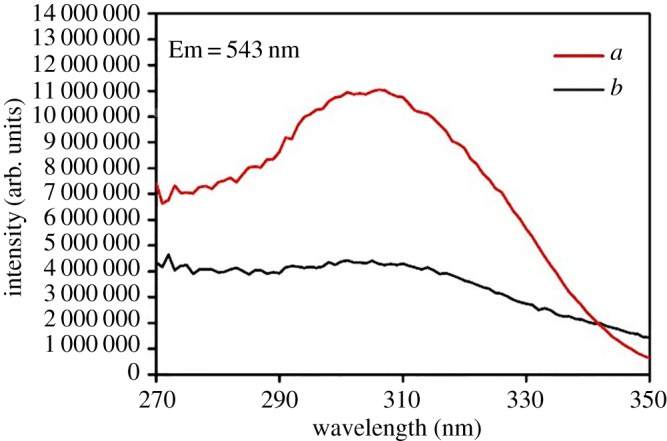
Excitation spectra of SiO_2(600)_@Tb(MABA-Si)·dipy (*a*) and Tb(MABA-Si)·dipy_2_·(ClO_4_)_3_·2H_2_O (*b*).

**Figure 10.**
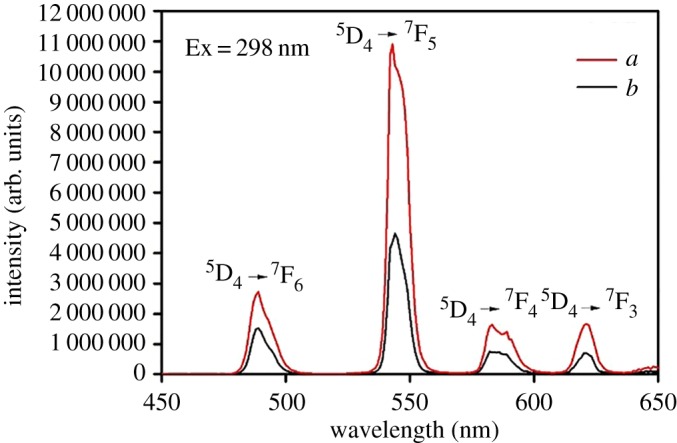
Emission spectra of SiO_2(600)_@Tb(MABA-Si)·dipy (*a*) and Tb(MABA-Si)·dipy_2_·(ClO_4_)_3_·2H_2_O (*b*).

**Figure 11.**
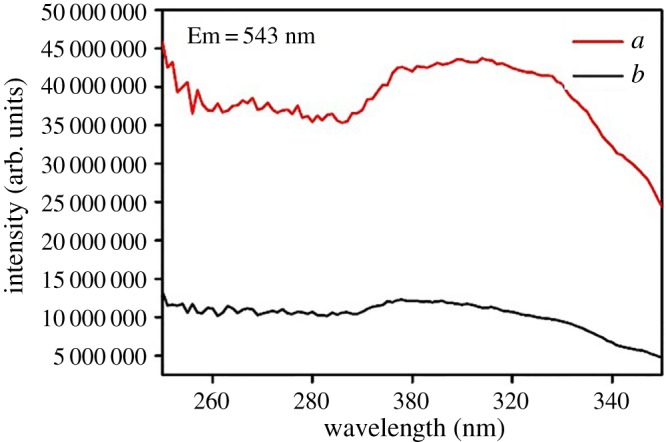
Excitation spectra of SiO_2(600)_@Tb(MABA-Si)·phen (*a*) and Tb(MABA-Si)·phen_2_·(ClO_4_)_3_·2H_2_O (*b*).

**Figure 12.**
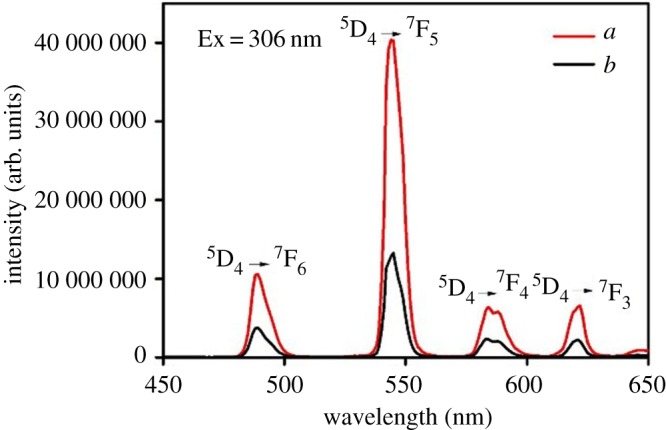
Emission spectra of SiO_2(600)_@Tb(MABA-Si)·phen (*a*), Tb(MABA-Si)·phen_2_·(ClO_4_)_3_·2H_2_O (*b*).

The luminescence properties of ternary terbium complexes and core–shell structure ternary terbium composites SiO_2(600)_@Tb(MABA-Si)·L have been investigated. The excitation spectra of Tb(MABA-Si)·dipy_2_·(ClO_4_)_3_·2H_2_O and SiO_2(600)_@Tb(MABA-Si)·dipy were measured by monitoring the emission of Tb^3+^ at 543 nm. As shown in figure 9, a broad excitation band extending from 200 to 400 nm presented the main peak centred at 298 nm. The corresponding emission spectra of the products are shown in figure 10. The emission peaks of the core–shell structure ternary terbium composite SiO_2(600)_@Tb(MABA-Si)·dipy and corresponding complex were located at 489, 543, 583 and 621 nm, which correspond to the ${\;}^{5}{\text{D}}_{4}\to^{7}{\text{F}}_{J}$ (*J* = 3–6) transitions of Tb^3+^ ions. The strongest emission peak located at 543 nm was attributed to ${\;}^{5}{\text{D}}_{4}\to^{7}{\text{F}}_{5}$ transitions of Tb^3+^ ions. The strongest emission intensity of SiO_2(600)_@Tb(MABA-Si)·dipy and Tb(MABA-Si)·dipy_2_·(ClO_4_)_3_·2H_2_O is 10 894 848 and 4 370 973 arb. units, respectively. It suggests that the very effective energy transfer from the ligand to Tb^3+^ ion in the complexes and core–shell structure ternary terbium composites and corresponding ternary terbium complexes. Two kinds of materials exhibit excellent characteristic green luminescence. It is worth noting that the core–shell structure ternary terbium composite SiO_2(600)_@Tb(MABA-Si)·dipy shows approximately 2.49 times stronger emission than the corresponding ternary terbium complex (table 3). Figure 11 presents typical excitation spectra of ternary terbium complex Tb(MABA-Si)·phen_2_·(ClO_4_)_3_·2H_2_O and core–shell structure ternary terbium composite SiO_2(600)_@Tb(MABA-Si)·phen. The obtained products possessed broad excitation bands with maxima at 306 nm and were recorded by monitoring the emission of Tb^3+^ ions at 543 nm. The emission spectra of SiO_2(600)_@Tb(MABA-Si)·phen and corresponding complex are shown in figure 12. SiO_2(600)_@Tb(MABA-Si)·phen and corresponding complex exhibited characteristic emission peaks of Tb^3+^ ${\;}^{5}{\text{D}}_{4}\to^{7}{\text{F}}_{J}$ (J = 6, 5, 4, 3) transition at 489 nm (${\;}^{5}{\text{D}}_{4}\to^{7}{\text{F}}_{6}$), 543 nm (${\;}^{5}{\text{D}}_{4}\to^{7}{\text{F}}_{5}$), 583 nm (${\;}^{5}{\text{D}}_{4}\to^{7}{\text{F}}_{4}$), 621 nm (${\;}^{5}{\text{D}}_{4}\to^{7}{\text{F}}_{3}$). The strongest emission peak located at 543 nm was attributed to ${\;}^{5}{\text{D}}_{4}\to^{7}{\text{F}}_{5}$ transitions of Tb^3+^ ions. The strongest emission intensity of Tb(MABA-Si)·dipy_2_·(ClO_4_)_3_·2H_2_O is 12 029 100 arb. units. It is more remarkable that strongest emission intensity of core–shell structure ternary terbium composite SiO_2(600)_Tb(MABA-Si)·phen is 40 339 128 arb. units, which is 3.35 times higher than that of the corresponding complex (table 3). The emission intensity of the obtained core–shell structure composites was increased, compared with the corresponding ternary complexes. We contribute this enhancement to the unique core–shell structure, in which the SiO_2_ cores greatly enhance the physical stability of ternary terbium complexes and decrease the energy loss of ternary terbium complexes molecular vibration. The luminescence emission intensity was increased. SiO_2_ core and organic ligands played a mutually synergistic part in the energy transfer process of the ligands to Tb^3+^ ions.

**Table 3.** Emission spectra data of the complexes and core–shell structure composites.

complexes	slit width (nm)	*λ*_EX_ (nm)	*λ*_EM_ (nm)	*I* (arb. units)	energy transition	intensity changes
Tb(MABA-Si)·phen_2_·(ClO_4_)_3_·2H_2_O	0.5	298	543	12 029 100	${\;}^{5}{\text{D}}_{4}\to^{7}{\text{F}}_{5}$	—
SiO_2(600)_@Tb(MABA-Si)·phen	0.5	298	543	40 339 128	${\;}^{5}{\text{D}}_{4}\to^{7}{\text{F}}_{5}$	3.35
Tb(MABA-Si)·dipy_2_·(ClO_4_)_3_·2H_2_O	0.5	306	543	4 370 973	${\;}^{5}{\text{D}}_{4}\to^{7}{\text{F}}_{5}$	—
SiO_2(600)_@Tb(MABA-Si)·dipy	0.5	306	543	10 894 848	${\;}^{5}{\text{D}}_{4}\to^{7}{\text{F}}_{5}$	2.49

To further discuss the luminescence properties of the resulting core–shell structure ternary terbium composites and corresponding complexes, the typical decay curves were measured. The resulting lifetime data of core–shell structure ternary terbium composites are given in table 4. The lifetime of the Tb^3+^ ions can be expressed by

3.1 $$I(t)=\;I_{0}+A_{1}\text{exp}\;\left(\frac{-t_{1}}{\tau_{1}}\right)+\;A_{2}\text{exp}\;\left(\frac{-t_{2}}{\tau_{2}}\right)$$

and

3.2 $$\langle \tau \rangle =\frac{\left(A_{1}{\tau_{1}}^{2}+\;A_{2}{\tau_{2}}^{2}\right)}{\left(A_{1}\tau_{1}+A_{2}\tau_{2}\right)},$$

where *I*(*t*) is the luminescence intensity varying with time *t*, and *τ*_1_ and *τ*_2_ are lifetime. The lifetimes of ternary terbium complex Tb(MABA-Si)·phen_2_·(ClO_4_)_3_·2H_2_O and corresponding composite are 0.57714 and 1.08274 ms, respectively. The lifetimes of ternary terbium complex Tb(MABA-Si)·dipy_2_·(ClO_4_)_3_·2H_2_O and corresponding composite are 1.03614 and 1.26420 ms, respectively. It is found that the core–shell structure ternary terbium composites present longer luminescent lifetimes than corresponding complex. It suggested that the rare earth complexes and core materials were connected by covalent bonds enhanced the luminescent stability.

**Table 4.** The lifetime of ternary terbium complexes and corresponding core–shell composites.

terbium complexes and core–shell composites	excited state	lifetime (ms)	*X*^2^
Tb(MABA-Si)·phen_2_·(ClO_4_)_3_·2H_2_O	^5^D_4_	0.57714	0.9993
SiO_2(600)_@Tb(MABA-Si)·phen	^5^D_4_	1.08274	0.9987
Tb(MABA-Si)·dipy_2_·(ClO_4_)_3_·2H_2_O	^5^D_4_	1.03614	0.9989
SiO_2(600)_@Tb(MABA-Si)·dipy	^5^D_4_	1.26420	0.9972

## 4. Conclusion

In this paper, two novel core–shell structure ternary terbium composites luminescent materials SiO_2(600)_@Tb(MABA-Si)·L were prepared by grafting the Tb(MABA-Si)·L_2_·(ClO_4_)_3_·2H_2_O complexes onto the surfaces of SiO_2_ core. In the reaction system, MABA-Si of Tb(MABA-Si)·L_2_·(ClO_4_)_3_·2H_2_O and SiO_2_ core formed Si–O–Si band by means of a molecule bridge that derived from the silylated hydrolysis and condensation. The luminescence properties indicate that core–shell structure ternary terbium composites SiO_2(600)_@Tb(MABA-Si)·L have stronger emission intensity than the corresponding complexes. And the core–shell structure ternary terbium composites have longer lifetimes. The formation of core–shell structure can increase the luminescence properties and reduce the amount of rare earth. This study provided new guidance in the design and fabrication of innovative rare earth materials. The synthesized novel core–shell structure terbium ternary composites have new opportunities to develop the application of rare earth element terbium.

## Data accessibility

The datasets supporting this article are available at the Dryad Digital Repository (http://dx.doi.org/10.5061/dryad.ht529) [38].

## References

[1] Runowski M, Grzyb T, Zep A, Krzyczkowska P, Gorecka E, Giersig M, Lis S 2014 Eu^3+^ and Tb^3+^ doped LaPO_4_ nanorods, modified with a luminescent organic compound, exhibiting tunable multicolour emission. RSC Adv. 4, 46 305–46 312. (doi:10.1039/c4ra06168c)

[2] Runowski M, Grzyb T, Lis S 2012 Magnetic and luminescent hybrid nanomaterial based on Fe_3_O_4_ nanocrystals and GdPO_4_:Eu^3+^ nanoneedles. J. Nanopart. Res. 14, 1188 (doi:10.1007/s11051-012-1188-7) 23087596 10.1007/s11051-012-1188-7 PMC3473191

[3] Dutta Choudhury S, Badugu R, Ray K, Lakowicz JR 2014 Surface-plasmon induced polarized emission from Eu(iii) – a class of luminescent lanthanide ions. Chem. Commun. 50, 9010 (doi:10.1039/c4cc03633f) 10.1039/c4cc03633f PMC4366936 24984065

[4] Yang Z, Kang S, Zhou R 2014 Nanomedicine: de novo design of nanodrugs. Nanoscale 6, 663–677. (doi:10.1039/c3nr04535h) 24305636 10.1039/c3nr04535h

[5] Chan CF et al. 2014 Bifunctional up-converting lanthanide nanoparticles for selective in vitro imaging and inhibition of cyclin D as anti-cancer agents. J. Mater. Chem. B 2, 84–91. (doi:10.1039/c3tb21034k) 10.1039/c3tb21034k 32261301

[6] Liang Y, Li Y, Wang H, Zhou J, Wang J, Regier T, Dai H 2011 Co_3_O_4_ nanocrystals on graphene as a synergistic catalyst for oxygen reduction reaction. Nat. Mater. 10, 780–786. (doi:10.1038/nmat3087) 21822263 10.1038/nmat3087

[7] Khan LU, Brito HF, Holsa J, Pirota KR, Muraca D, Felinto MCFC, Teotonio EES, Malta OL 2014 Red-green emitting and superparamagnetic nanomarkers containing Fe_3_O_4_ functionalized with calixarene and rare earth complexes. Inorg. Chem. 53, 12 902–12 910. (doi:10.1021/ic5018856) 25475194 10.1021/ic5018856

[8] Fleaca CT, Dumitrache F, Morjan I, Niculescu AM, Sandu I, Ilie A 2016 Synthesis and characterization of polyaniline–Fe@C magnetic nanocomposite powder. Appl. Surf. Sci. 374, 213–221. (doi:10.1016/j.apsusc.2015.11.043)

[9] Qiu P, Zhou N, Chen H, Zhang C, Gao G, Cui D 2013 Recent advances in lanthanide-doped upconversion nanomaterials: synthesis, nanostructures and surface modification. Nanoscale 5, 11 512–11 525. (doi:10.1039/c3nr03642a) 10.1039/c3nr03642a 24121736

[10] Nagabhushana H, Sunitha DV, Sharma SC, Prashantha SC, Nagabhushana BM, Chakradhar RPS 2014 CdSiO_3_:Eu^3+^, red nanophosphors prepared by low temperature solution combustion technique, its structural and luminescent properties. J. Alloy. Compd. 616, 284–292. (doi:10.1016/j.jallcom.2014.05.228)

[11] Ishii H, Kawai S, Nagao D, Konno M 2015 Synthesis of phosphor-free luminescent, monodisperse, mesoporous silica nanoparticles in the co-presence of double- and single-chain cationic surfactants. Adv. Powder Technol. 27, 448–453. (doi:10.1016/j.apt.2016.01.010)

[12] Li H, Kang J, Yang J, Wu B 2016 Distance dependence of fluorescence enhancement in Au nanoparticle@mesoporous silica@europium complex. J. Phys. Chem. C 120, 16 907–16 912. (doi:10.1021/acs.jpcc.6b01312)

[13] Wei W, Zhao Y, Peng S, Zhang H, Bian Y, Li H, Li H 2014 Yolk-shell nanoarchitectures with a Ru-containing core and a radially oriented mesoporous silica shell: facile synthesis and application for one-pot biomass conversion by combining with enzyme. ACS Appl. Mater. Interfaces 6, 20 851–20 859. (doi:10.1021/am5052608) 10.1021/am5052608 25405326

[14] Chowdhury S, Wu Z, Jaquinsgerstl A, Liu S, Dembska A, Armitage BA, Rongchao J, Linda AP 2011 Wavelength dependence of the fluorescence quenching efficiency of nearby dyes by gold nanoclusters and nanoparticles: the roles of spectral overlap and particle size. J. Phys. Chem C 115, 20 105–20 112. (doi:10.1021/jp204836w) 10.1021/jp204836w PMC3424614 22924090

[15] Singh LP, Srivastava SK, Mishra R, Ningthoujam RS 2014 Multifunctional hybrid nanomaterials from water dispersible CaF_2_:Eu^3+^, Mn^2+^ and Fe_3_O_4_ for luminescence and hyperthermia application. J. Phys. Chem. C 118, 18 087–18 096. (doi:10.1021/jp502825p)

[16] Shi D, Sadat ME, Dunn AW, Mast DB 2015 Photo-fluorescent and magnetic properties of iron oxide nanoparticles for biomedical applications. Nanoscale 7, 8209–8232. (doi:10.1039/c5nr01538c) 25899408 10.1039/c5nr01538c

[17] Li MJ, Chen Z, Yam VW, Zu Y 2008 Multifunctional ruthenium(II) polypyridine complex-based core-shell magnetic silica nanocomposites: magnetism, luminescence, and electrochemiluminescence. ACS. Nano. 2, 905–912. (doi:10.1021/nn800123w) 19206487 10.1021/nn800123w

[18] Debasu ML, Ananias D, Pastoriza-Santos I, Liz-Marzán LM, Rocha J, Carlos LD 2013 All-in-one optical heater-thermometer nanoplatform operative from 300 to 2000 K based on Er(^3+^) emission and blackbody radiation. Adv. Mater. 25, 4868–4874. (doi:10.1002/adma.201300892) 23696297 10.1002/adma.201300892

[19] Wei S, Wang Q, Zhu J, Sun L, Lin H, Guo Z 2011 Multifunctional composite core-shell nanoparticles. Nanoscale 3, 4474–4502. (doi:10.1039/c1nr11000d) 21984390 10.1039/c1nr11000d

[20] Li WX, Zheng YS, Cao XF, Bai J, Fu ZF, Bao JR, Li YL 2016 Preparation, characterization, and luminescence properties of dysprosium perchlorate with MABA-Si and phen or dipy complexes as well as SiO_2_@Dy(MABA-Si)L core-shell structure nanometermeter luminescent composites. J. Lumin. 178, 470–478. (doi:10.1016/j.jlumin.2016.06.019)

[21] Ghosh Chaudhuri R, Paria S 2012 Core/shell nanoparticles: classes, properties, synthesis mechanisms, characterization, and applications. Chem. Rev. 112, 2373–2433. (doi:10.1021/cr100449n) 22204603 10.1021/cr100449n

[22] Ansari AA, Hasan TN, Syed NA, Labis JP, Parchur AK, Shafi G, Alshatwi AA 2013 In-vitro cyto-toxicity, geno-toxicity, and bio-imaging evaluation of one-pot synthesized luminescent functionalized mesoporous SiO_2_@Eu(OH)_3_ core-shell microspheres. Nanomedicine 9, 1328–1335. (doi:10.1016/j.nano.2013.05.006) 23727099 10.1016/j.nano.2013.05.006

[23] Liu C, Yan B 2015 Highly effective chemosensor of a luminescent silica@lanthanide complex@MOF heterostructured composite for metal ion sensing. Rsc Adv. 5, 101 982–101 988. (doi:10.1039/c5ra19973e)

[24] Liu M, Gan L, Chen L, Xu Z, Zhu D, Hao Z, Chen L 2012 Supramolecular core-shell nanosilica@liposome nanocapsules for drug delivery. Langmuir 28, 10 725–10 732. (doi:10.1021/la3021645) 22746205 10.1021/la3021645

[25] Wang QM, Yan B 2004 Novel luminescent terbium molecular-based hybrids with modified m-aminobenzoic acid covalently bonded with silica. J. Mater. Chem. 14, 2450–2454. (doi:10.1039/b402667e)

[26] Li HR, Lin J, Zhang HJ, Fu LS, Meng QG, Wang SB 2002 Preparation and luminescence properties of hybrid materials containing europium(III) complexes covalently bonded to a silica matrix. Chem. Mater. 14, 3651–3655. (doi:10.1021/cm0116830)

[27] Davies GL, Barry A, Gunko YK 2009 Preparation and size optimisation of silica nanoparticles using statistical analyses. Chem. Phys. Lett. 468, 239–244. (doi:10.1016/j.cplett.2008.12.031)

[28] Fielding LA, Tonnar J, Armes SP 2011 All-acrylic film-forming colloidal polymer/silica nanocomposite particles prepared by aqueous emulsion polymerization. Langmuir 27, 11 129–11 144. (doi:10.1021/la202066n) 21776995 10.1021/la202066n

[29] Geary GW 1971 α-Haloalkyl and related reagents. Their preparation and synthetic utility. J. Coord. Chem. Rev. 7, 81–94. (doi:10.1016/s0010-8545(00)80009-0)

[30] Qin C, Wang XL, Wang AE, Su ZM 2005 A series of three-dimensional lanthanide coordination polymers with rutile and unprecedented rutile-related topologies. Inorg. Chem. 44, 7122–7129. (doi:10.1021/ic050906b) 16180874 10.1021/ic050906b

[31] Petrov VA, Marshall WJ, Grushin VV 2002 The first perfluoroacetylacetonate metal complexes: as unexpectedly robust as tricky to make. Chem. Commun. 5, 520–521. (doi:10.1039/b111249j) 10.1039/b111249j 12120570

[32] Cho J, Lough AJ, Ju CK 2003 Monomeric and polymeric copper(II) hexaaza macrocyclic complexes with btc anions (btc = 1,2,4,5-benzenetetracarboxylic acid). Inorg. Chim. Acta 342, 305–310. (doi:10.1016/s0020-1693(02)01149-0)

[33] Crutchley RJ, Lever ABP 1982 Comparative chemistry of bipyrazyl and bipyridyl metal complexes: spectroscopy, electrochemistry and photoanation. Inorg. Chem. 21, 2267–2282. (doi:10.1021/ic00136a030)

[34] Qian GD, Wang MQ, Lv SZ, Chin. 1998 Synthesis characterization and fluorescence of Eu^3+^, Tb^3+^ complexes with heterocyclic ligands containing nitrogen. Chinese J. Lumin 19, 60–65. (http://kns.cnki.net/KCMS/detail/detail)

[35] Xu CJ, Chin J 2006 Synthesis and photoluminescence properties of Eu(Gd) complexes with salicylic acid and o-phenanthroline. Rare Earth Soc, 24, 361 (http://kns.cnki.neSldens&v)

[36] Rosenthal MR 1973 Myth of the noncoordinating anion. J. Chem. Educ. 50, 331–335. (doi:10.1021/ed050p331)

[37] Hathaway BJ, Underhill AE 1961 The infrared spectra of some transition-metal perchlorates. J. Chem. Soc. 65, 3091–3096. (doi:10.1039/jr9610003091)

[38] Ma Y-Y, Li W-X, Zheng Y-S, Bao J-R, Li Y-L, Feng L-N, Yang K-S, Qiao Y, Wu A-P 2018 Data from: Preparation, characterization and luminescence properties of core–shell ternary terbium composites SiO_2(600)_@Tb(MABA-Si)•L Dryad Digital Repository. (doi:10.5061/dryad.ht529) 10.1098/rsos.171655 PMC5882697 29657773

